# Current Insights in Bidirectional Cardio-Oncology: Heart Failure Driving Cancer: *JACC: CardioOncology* Short-Form Primer

**DOI:** 10.1016/j.jaccao.2025.05.014

**Published:** 2025-08-19

**Authors:** Rudolf A. de Boer, Laura I. Yousif, Joseph Pierre Aboumsallem, Wouter C. Meijers

**Affiliations:** Department of Cardiology, Cardiovascular Institute, Thorax Center, Erasmus Medical Center, Rotterdam, the Netherlands

**Keywords:** cancer, cardiovascular disease, heart failure, mechanisms, reverse cardio-oncology, risk factor, underlying mechanisms

## Abstract

• The presence and pathophysiological correlations of HF increase risk for cancer.
• HF-GRMT does not have a clear effect on incident cancer.
• Preclinical cardio-oncology models reveal a complex and multifactorial relationship.
• Clinical research can identify circulating factors as biomarkers/therapeutic targets.

## Graphical Abstract

**Figure:**
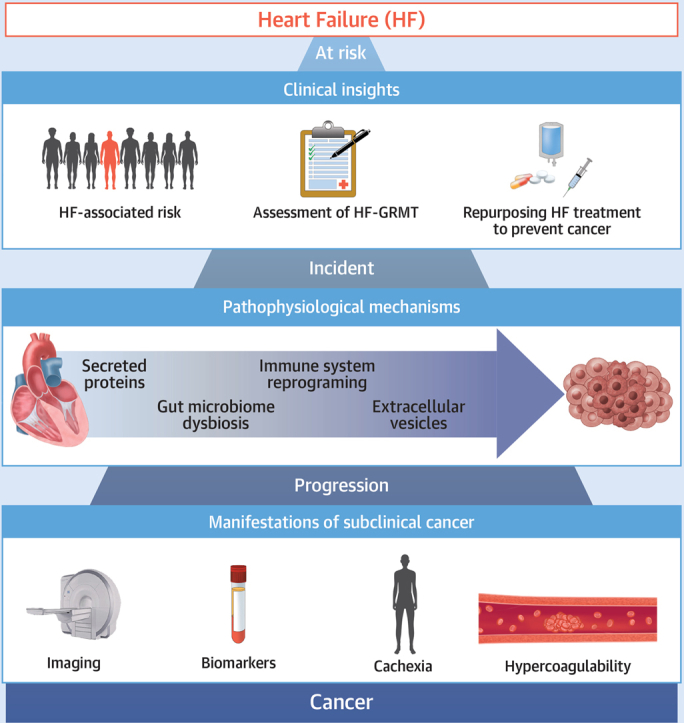

It is well established that individuals undergoing cancer treatment are at risk for cardiovascular disease (CVD), including heart failure (HF). However, as the field of cardio-oncology has grown, research into cardiotoxicity mechanisms has highlighted shared risk factors and underlying mechanisms connecting HF and cancer, spurring on scientists to investigate the “reverse” relationship between CVD and cancer. Indeed, a number of recent publications have highlighted a higher rate of incident cancer and cancer-related mortality in patients with HF compared with those without HF.^1^ As a result, a classification was proposed to describe this and various other cardio-oncology syndromes, in which cancer may precede or follow HF and may be a direct or indirect effect (eg, via drugs).^2^ In this short primer, we provide a brief and timely update on the latest scientific insights on HF-associated cancer incidence and growth.

## Clinical Insights on HF-Driven Cancer

Although the relationship between HF and increased cancer incidence has been consistently observed, the underlying mechanisms remain largely unknown. In a general population cohort, HF had an attributable risk of 16% for the development of cancer after adjustment for relevant known shared risk factors of CVD and cancer (eg, age, sex, tobacco use and alcohol assumption).^3^ This suggests that additional drivers of cancer in individuals with CVD may exist. Regarding site-specific cancers, a significant risk for cancer was observed for many solid tumors, but especially colorectal cancer (CRC), lung cancer, and multiple myeloma. These findings advocate for greater and continued transdisciplinary collaborations between cardiologists and oncologists.

Given the relationship between HF and cancer, the question has been raised whether guideline-directed medical therapies for HF may (in part) explain this interaction. In a recent appraisal of preclinical and clinical data, it was concluded that the use of contemporary guideline-recommended medical therapies (eg, beta-blockers, renin-angiotensin-aldosterone inhibitors, mineralocorticoid receptor antagonists and sodium-dependent glucose cotransporter 2 inhibitors) was not associated with an increased rate of all-cause or site-specific cancer.^4^ Although additional (long-term) follow-up is needed to generate more robust data, on the basis of the current literature, guideline-directed medical therapy for HF does not appear to be integral in HF-mediated cancer incidence. However, treatment modalities used in CVD and HF are being evaluated for antitumor effects. The widespread beneficial effects of sodium-dependent glucose cotransporter 2 inhibitors have prompted interest in noncardiovascular outcomes, and their use has been associated with a reduction in incident cancers in several analyses.^2^^,^^5^ Similarly, glucagon-like peptide 1 receptor analogues, indicated for type 2 diabetes mellitus and obesity, were associated with both reductions of cardiovascular-related morbidity and mortality, and various forms of cancer, especially liver cancer.^6^ Besides medication, the potential of treatment modalities for unexplored shared risk factors has been considered. In HF as well as cancer, obesity is associated with factors triggering inflammation and other detrimental factors (eg, oxidative stress).^2^ For instance, maintenance of a healthy lifestyle and weight is known to contribute to cardiovascular health, but recently bariatric surgery has also been associated with a reduced incidence of hematological cancers and cancer-related mortality, specifically in women.^7^ These clinical insights fill gaps in knowledge in clinical practice. But they also raise questions that should be addressed by mechanistic studies.

## Proposed Pathophysiological Mechanisms Behind HF-Induced Tumor Growth

A study by Meijers et al^8^ showed, for the first time, a direct causal effect of post–myocardial infarction HF on tumor growth, independent of hemodynamic status, which was attributed to specific circulating factors (most notably SerpinA3). Since then, multiple studies in various HF models have corroborated this initial finding, identifying several factors as potential culprits (Table 1).

**Table 1:** Overview of Mechanistic Models of Reverse Cardio-Oncology

Linking Mechanism	Observation	Mechanistic Insight
Secreted factors		
Circulating proteins	• Myocardial infarction stimulated polyp growth in APCmin mice • High levels of SerpinA3 were detected in serum of patients with chronic HF • Pressure overload–induced HF caused larger primary breast and lung cancer in C57Bl/6 mice	• Increased levels of cardiac-secreted SerpinA3 associated with tumor growth • Cardiac fibroblast–secreted periostin was implicated
Extracellular vesicles	• Uptake of cMSC-EVs from mice with myocardial infarction accelerated tumor growth in vitro and in vivo in an adoptive transfer model	• cMSC-EVs from mice with myocardial infarction showed high levels of protumorigenic factors in their cargo
Gut microbiome	• HF was associated with a dysregulated microbiome, gut congestion, and low-grade inflammation • Microbial dysbiosis was associated with colorectal cancer • Myocardial infarction increased colonic tumor formation in mice	• Microbial dysbiosis induced by myocardial infarction resulted increased colonic tumor formation in a fecal transplantation model
Immune regulatory processes	• Breast cancer growth was accelerated in mice with a myocardial infarction in the circulation	• Enhanced proinflammatory and protumorigenic Ly6Chi monocytes drove tumor growth and targeted depletion of these cells abrogated tumor growth

cMSC-EV = cardiac small mesenchymal stromal cell–derived extracellular vesicle; HF = heart failure.

Pressure overload–induced HF was mimicked though transverse aortic constriction (TAC) in mice, which were also inoculated with breast or lung cancer cells.^9^ TAC mice showed larger primary tumors and a higher metastatic seeding rate compared with control mice. Through RNA sequencing, periostin, a protein secreted mainly by activated fibroblasts and a major contributor to extracellular matrix remodeling in the heart and in tumors, was identified as a possible culprit. In vitro stimulation of breast and lung cancer cells with periostin caused dose-dependent cell proliferation. Full serum from TAC mice also increased cell proliferation, whereas periostin-depleted serum did not.

An entirely different source of circulating factors are cardiac-derived extracellular vesicles (EVs), which potentially could also exert tumor-promoting effects. Indeed, recently, it was shown that cardiac small mesenchymal stromal cell-derived EVs (cMSC-EVs) isolated from infarcted hearts of mice contain unique protein profiles with high quantities of tumor-promoting cargo.^10^ In vitro stimulation of tumor cells from various origins with these cMSC-EVs showed varying degrees of responses, including the ability to polarize resting macrophages to a protumorigenic phenotype. Additionally, adoptive transfer of cMSC-EVs from murine infarcted hearts accelerated growth of heterotopic and orthotopic lung tumors, whereas depletion significantly reduced the tumor-promoting effects. Another potential link between HF and cancer is the gut microbiome. HF is characterized by specific dysregulation of the gut microbiome, which is associated with gut congestion and low-grade inflammation. At the same time, there is (pre)clinical evidence linking dysbiosis of the microbiome to CRC. In a recent study in mice with HF, the gut microbiome was mapped^11^ and microbial dysbiosis was demonstrated (evidenced by reduced α-diversity and genus-level alterations). These changes mimicked microbial changes that previously had been associated with CRC. Upon transplantation of fecal matter from control and HF mice into a germ-free CRC mouse model, an increase in colonic tumor formation was observed in response to fecal matter with HF-mediated gut dysbiosis. This mechanism presents another layer in the relationship between incident cancer and HF and adds support to the clinical finding that CRC is one of the most prominent cancers in patients with HF.^3^

Finally, another study in mice with myocardial infarction demonstrated that accelerated breast cancer growth was markedly associated with the influx of CD11b^+^Ly6Chi monocytes, which became tumor-promoting, immunosuppressive macrophages in the tumor microenvironment.^12^ Targeted depletion of these cells abrogated tumor growth and suggest that enhanced Ly6Chi monocytes are crucial to tumor growth in this setting.

The relationship between HF and cancer is thus pleiotropic and multifactorial. We are currently unaware of data detailing the relative contributions of the proposed mechanisms in each of the various HF etiologies across multiple cancers.

## Manifestations of Subclinical Incident Cancer

Another factor to consider is that signs of an unmasked or potentially subclinical incident cancer may be present upon diagnosis or during treatment of HF.^2^ This warrants caution because it implies that cancer in this population of patients could potentially be (pre-emptively) detected, diagnosed, and treated. There is overlap in symptoms between CVD and cancer (eg, fatigue, dyspnea, mild anemia), and caution should be exercised before labeling these symptoms as cardiac specific. For example, many patients with HF have anemia. This often is explained by chronic disease and iron deficiency; however, given that anemia is also a symptom of cancer, this maybe overlooked. CVD diagnostic tools have been found to hold predictive value for incident cancer (eg, galectin-3).^13^ Patients with HF also have elevated levels of biomarkers that are linked with processes commonly considered “hallmarks of cancer,” such as cachexia and wasting, and all-cause mortality.^14^ This further warrants multidisciplinary assessment of patients with HF. In addition, patients with HF frequently undergo medical imaging studies, which may provide clues for (concealed) cancer. Finally, other HF-related phenomena may contribute to HF-associated cancer growth, such as hypoxia and hypercoagulability. Hypoxia is commonly characterized by an increase in the protein hypoxia-inducible factor 1-alpha, which is increased in various forms of CVD and HF, and several cancers overexpress hypoxia-inducible factor 1-alpha.^2^ Patients with HF also often present with a hypercoagulable state because of conditions such as decreased blood flow (stasis) or endothelial cell dysfunction (Virchow’s triad).^2^ Coagulant factors (eg, thrombin) have been suggested to promote cancer metastasis and induce vascular growth factors and angiogenesis.

## Conclusions and Implications

**Figure 1:**
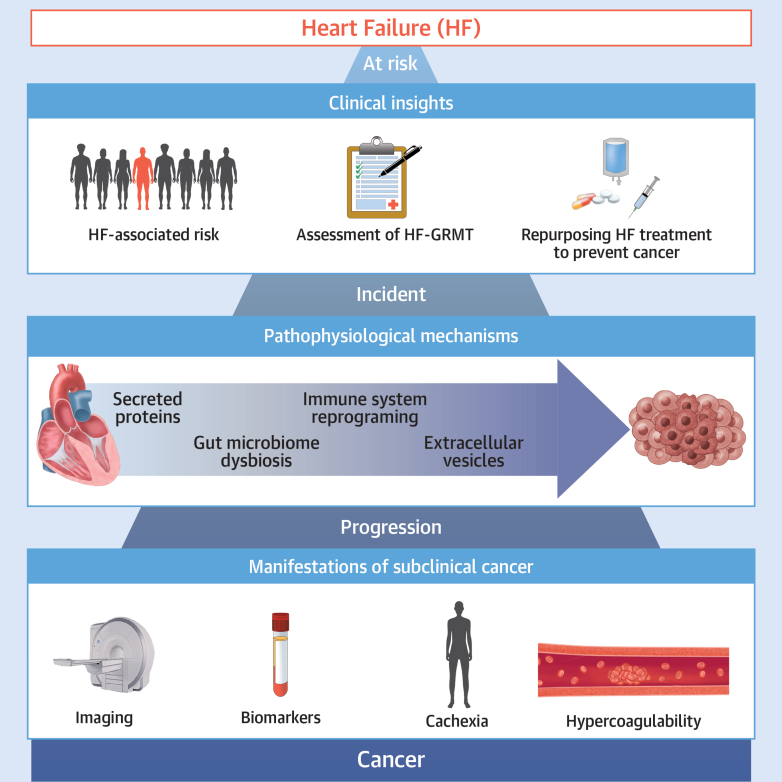
Crosstalk Between HF and Cancer Throughout the Spectrum The relationship between heart failure (HF) and cancer is being investigated in each phase of the spectrum; clinical insights suggest that patients with HF might be more at risk of developing specific cancers and that HF treatment might be beneficial; during incident HF, there are multiple pathophysiological mechanisms that can drive tumor growth (eg, secreted proteins, the gut microbiome), and certain clinical manifestations of progressed HF can unmask the presence of already existing cancer (eg, hypercoagulability, certain biomarkers). GRMT = guideline-recommended medical therapy.

This field of reverse, or bidirectional, cardio-oncology is rapidly progressing. The presence of HF by virtue of pathophysiological mechanisms of HF gives rise to an increased risk for incident cancer. The complex nature results in a variety of factors and potential underlying mechanisms, which requires further investigation with comprehensive preclinical and clinical studies to map the full framework of interactions (Figure 1). Key topics for future research should aim to unravel if shared factors are amenable to treat both HF and cancer simultaneously and whether (circulating) factors may be insightfully used to detect unknown, subclinical disease.

## Funding Support and Author Disclosures

Dr de Boer is supported by the Netherlands Heart Foundation (grants 2020B005 and 01-003-2022-0358), a Netherlands Heart Foundation grant cofunded by ERA4Health (GA grant 101095426 of the European Union Horizon Europe Research and Innovation Programme), and the European Research Council (CoG 818715). Dr Meijers is supported by grants from the Dutch Heart Foundation (Dekkerbeurs 03-005-2021-T005) and ZonMw (Off Road [04510012210034] and NWO-VENI [09150162310159]). The institution of Dr de Boer has received research grants and/or fees from Alnylam, AstraZeneca, Abbott, Bristol Myers Squibb, Novo Nordisk, and Roche. Dr de Boer has had speaker engagements with, received fees from, and/or served on advisory boards for Abbott, AstraZeneca, Bristol Myers Squibb, Novo Nordisk, Roche, and Zoll; and has received travel support from Abbott and Novo Nordisk. Dr Meijers has received speaker and advisory board fees from Daiichi-Sankyo and Novartis. All other authors have reported that they have no relationships relevant to the contents of this paper to disclose.
